# Impaired Mitochondrial Function Results from Oxidative Stress in the Full-Term Placenta of Sows with Excessive Back-Fat

**DOI:** 10.3390/ani10020360

**Published:** 2020-02-23

**Authors:** Liang Tian, Jiahe Huang, Aiyou Wen, Peishi Yan

**Affiliations:** 1 College of Animal Science and Technology, Nanjing Agricultural University, Nanjing 210095, China; 2 College of Animal Science and Technology, Northwest A & F University, Yangling 712100, China; 3 College of Animal Science, Anhui Science and Technology University, Fengyang 233100, China

**Keywords:** NF-κB, mitochondria, oxidative stress, back-fat, placenta, pig

## Abstract

**Simple Summary:**

Placental dysfunction associated with maternal obesity has been demonstrated to be a possible detrimental determinant for a reproductive disorder in human and animals such as pigs. Moreover, there exists a substantial amount of evidence supporting that mitochondrial dysfunction associated with obesity contributes to dysfunction of highly metabolic tissues, including adipose, skeletal muscle and placenta. Despite previous reports have demonstrated that back-fat thickness of sows is associated with placental dysfunction, the influences of excessive back-fat on mitochondrial structure and function in porcine placenta still remain elusive. In this study, animal (Landrace) and cell in vitro model (pig placental trophoblasts) were employed to evaluate mitochondrial alterations in the placentas of sows with different back-fat depth. We revealed that excessive back-fat of sows is associated with placental mitochondrial abnormalities corresponding to decreased ATP production and impaired mitochondrial respiration in the placenta. Together, our findings develop the understanding about the impact of excessive back-fat induced oxidative stress on mitochondrial alterations in the pig placenta, which may contribute to generate some strategies in future to improve sow reproduction.

**Abstract:**

The aim of this study was to determine the effect of excessive back-fat (BF) of sows on placental oxidative stress, ATP generation, mitochondrial alterations in content and structure, and mitochondrial function in isolated trophoblasts. Placental tissue was collected by vaginal delivery from BFI (15–20 mm, n = 10) and BFII (21–27 mm, n = 10) sows formed according to BF at mating. Our results demonstrated that excessive back-fat contributed to augmented oxidative stress in term placenta, as evidenced by excessive production of ROS, elevated protein carbonylation, and reduced SOD, GSH-PX, and CAT activities (*p* < 0.05). Indicative of mitochondrial dysfunction, reduced mitochondrial respiration in cultured trophoblasts was linked to decreased ATP generation, lower mitochondrial Complex I activity and reduced expression of electron transport chain subunits in placenta of BFII sows (*p* < 0.05). Meanwhile, we observed negative alterations in mitochondrial biogenesis and structure in the placenta from BFII group (*p* < 0.05). Finally, our in vitro studies showed lipid-induced ROS production resulted in mitochondrial alterations in trophoblasts, and these effects were blocked by antioxidant treatment. Together, these data reveal that excessive back-fat aggravates mitochondrial injury induced by increased oxidative stress in pig term placenta, which may have detrimental consequences on placental function and therefore impaired fetal growth and development.

## 1. Introduction

Pregnancy complicated by maternal obesity is associated with an abnormal intrauterine milieu characterized by increased lipid accumulation, augmented oxidative stress, and significant inflammation within the placenta [1,2], resulting in placental dysfunction and therefore the poor pregnancy outcomes in human beings and animals such as pig [3,4]. Excessive lipid accumulation has been shown to induce cellular dysfunction through the over production of reactive oxygen species (ROS), mitochondrial dysfunction, and the activation of inflammation in highly metabolic tissues like adipose and skeletal muscle [5,6]. As an extremely metabolically active fetal tissue, the placenta is also susceptible to obesity-associated lipotoxicity [1,7]. Recently, lipotoxicity has been demonstrated to inhibit placental development and induce placental dysfunction evidenced by dysregulation of lipid metabolism and transport in the human or pig full-term placenta [8,9,10].

As a significant marker of placental lipotoxicity, oxidative stress can be increased in pregnancy complicated by maternal obesity resulting in placental oxidative injure [11]. In addition, mitochondria, a key energy source for placental function, are also the major source of ROS under physiologic conditions. However, mitochondrial function itself can be compromised by severe or prolonged oxidative stress [12]. Excessive ROS can damage lipids, proteins, and nucleic acids within the mitochondria, leading to changes in mitochondrial structure and function [13]. Several studies have shown that increased metabolic activity of placental mitochondria and the reduced antioxidant scavenging power may be responsible for increased ROS generation and lower mitochondrial respiration in pregnancies complicated by maternal obesity or diabetes, resulting in detrimental consequences on placental function and therefore fetal developmet [14,15]. Thus, mitochondrial dysfunction and over generation of superoxide could be part of a vicious cycle and can be considered as a potential mechanism of placental dysfunction involved in obese pregnancy. Although several studies reveal that excessive back-fat of sows is associated with placental dysfunction [4,16], it is not currently known whether increased back-fat is coupled with changes in mitochondrial structure and function in the pig placenta.

In this study, we addressed the hypothesis that increased back-fat affect mitochondrial structure and function in porcine placenta. Thus, the purpose of this study was to investigate the effect of excessive back-fat of sows on placental ROS production, mitochondrial biogenesis, mitochondrial respiration, and mitochondria alterations in density and structure. In addition, we assessed the role of lipid-induced oxidative stress in the development of mitochondrial abnormalities in pig placental trophoblast cells in vitro and found that lipid-induced ROS production resulted in mitochondrial alterations in trophoblasts. Consistent with our hypothesis, we demonstrated that excessive back-fat resulted in increased ROS production, reduced mitochondrial biogenesis, impaired mitochondrial respiration, and mitochondrial abnormalities in content and structure in pig full-term placenta, suggesting excessive back-fat of sows may promote placental mitochondrial dysfunction that is associated with impaired placental function and fetal development.

## 2. Materials and Methods

### 2.1. Animal Model and Dietary Management

All animal procedures involved in this study were specifically approved by the Laboratory Animal Care and Use Committee of Nan Jing Agricultural University (SYXK2015-0072, 6 September 2015). A total of 20 lean breed sows (Landrace) were investigated in the present study. All the sows were multiparous with same parity (Parity = 2) and inseminated with pooled semen doses containing 3–4 billion sperm cells to produce purebred litters. After the weaning period with a corn soybean diet (3200 Kal/kg of metabolizable energy, 18% of crude protein and 0.9% lysine), the females were dichotomized based on BF (back-fat thickness) at mating into BFI (15–20 mm, n = 10) and BFII (21–27 mm, n = 10) as described previously [17]. All the sows were housed together in passively ventilated collective pens with concrete slatted floors at the Research Farm of Nan Jing Agricultural University. During pregnancy, a standard grain-based diet (89.9% of dry matter, 14.5% of crude protein, 3300 Kcal/kg of metabolizable energy) was offered per day, divided over two meals with ad libitum access to water. The amount of food offered to each sows was adjusted to 1.9, 2.2, 2.4, and 2.9 kg of feed per day at 0–30, 31–60, 61–90, and 90–110 days of gestation (day 0 = day of insemination), respectively, based on the data from the National Research Council (NRC) (2012).

### 2.2. Data Collection and Sampling

Sows in this study were weighted and measured for back-fat thickness at mating (2 to 3 days before insemination) and at farrowing, respectively, by the research team with the help of farm personnel. Back-fat depth (BF) was determined by A-mode ultrasonography (Renco, Rockledge, FL, USA) as previously described [18]. Briefly, the probe was placed in a point at the right side of the animal located at 4 cm from the midline and transversal to the head of the last rib as determined by palpation. At day 105 of pregnancy, maternal blood (overnight fasting) was collected into 5-mL sterile heparinized vacuum tubes (Greiner, Frickenhausen, German). Immediately after recovery, blood samples were centrifuged at 3500× *g* for 15 min, and the plasma was separated and stored at −80 °C for further analysis. After removal of fetal amnion, placental villous tissues, obtained from vaginal delivery, were rinsed thoroughly in cold Phosphate-buffered saline (PBS) solution before further processing. Placental samples were then cut into approximately 5-cm^2^ pieces and flash-frozen in liquid nitrogen and stored at −80 °C until further processing. For each pregnancy, piglets born alive and stillborn were counted and individually weighed within 12 h after birth, and the placentas were also weighed for measuring placental efficiency as a ratio of litter weight to placental weight as previously described [10].

### 2.3. Cell Culture and Drug Treatment

Porcine placental trophoblast cells (cytotrophoblasts) were isolated from fresh porcine full-term placentas, as previously described [19]. Briefly, placental tissue, obtained from vaginal delivery, was separated from the visible blood vessels as well as connective tissues, thoroughly washed with cold PBS containing 100 U/mL penicillin (Invitrogen, Carlsbad, CA, USA) and 100 μg/mL streptomycin (Invitrogen, Carlsbad, CA, USA), and then excised and cut into 1–3 mm^3^ pieces. The tissue fragments were digested three times at 37 °C for 30 min with continuous shaking, followed by filtration through a 70 μm cell strainer. The digestion medium was composed of Ham’s F12/Dulbecco’s Modified Eagle Medium (DMEM/F12) (HyClone, Logan, UT, USA), 0.125% (*W*/*V*) trypsin (Gibco, Grand Island, NY, USA), 20 U/mL DNase I (Roche, Basel, Switzerland), and 0.1% BSA (Amresco, Solon, OH, USA). The cell suspension of all three digestions was centrifuged at 1000× *g* for 10 min to collect the cell pellet. Pellets were then resuspended in DMEM/F12 and deposited on top of a discontinuous 35% and 45% (*V*/*V*) Percoll (Pharmacia, London, UK) gradient solution, and centrifuged at 2000× *g* for 20 min. Villous cytotrophoblasts were collected from the appropriate layers, and cultured in DMEM/F12 supplemented with 10% FBS (HyClone, Logan, UT, USA), 1% (*V*/*V*) Insulin–Transferrin–Selenium (ITS; Sigma, Saint Louis, MO, USA), 10 ng/mL of epidermal growth factor (EGF; Invitrogen, Carlsbad, CA, USA), 100 U/mL penicillin, and 100 μg/mL streptomycin at 37 °C under 5% CO_2_ as previously reported [19,20]. The purity of preparations of cytotrophoblasts isolated from full-term placentas was evaluated by flow cytometry as previously described [20], using FITC fluorescein-labeled antibody against cytokeratin-7 (Santa Cruz Tech, Dallas, CA, USA) as a specific marker of trophoblast cells.

In order to induce oxidative stress in cytotrophoblasts in vitro, cells were treated with 400 μM Fatty Acid (FA) Supplement containing 2 mol of linoleic acid and 2 mol of oleic acid per mole of albumin (L9655; Sigma-Aldrich, St. Louis, MO, USA) in triplicate as previously described [19,21]. Treatment media without fatty acids was added with bovine serum albumin (FA free) to maintain the same osmolarity. In cell experiments in vitro, cells were treated with 400 μM FA or 2 mM Vitamin E (VE) (oxidative stress inhibitor; MCE, Shanghai, China) for the amount of time specified in the individual figures.

### 2.4. Plasma Assay

The concentrations of triglyceride (TG) and nonesterified fatty acids (NEFA) in plasma samples were determined by using Beckman AU5811 analyzer (Beckman-Coulter, Inc.250S.Kraemer Boulevard Brea, CA, USA) as previously reported [8]. A commercially available porcine specific ELISA kit (RD SYSTEMS, Minneapolis, MN, USA) and an Amplex Red hydrogen peroxide assay kit (Invitrogen, Carlsbad, CA, USA) were utilized to measure plasma leptin and hydrogen peroxide (H_2_O_2_) content, respectively, according to the manufacturer’s instructions as previously described [5,17]. All blood samples were determined in duplicate in single assay. The minimum detection limit was 3.2 pg/dL, 0.1 pmol/L, 0.2 ng/mL, and 0.2 pmol/L for TG, NEFA, Leptin, and H_2_O_2_, respectively. For Leptin and H_2_O_2_ analysis, within-assay coefficient of variation (CV) was acceptable when less than 5%.

### 2.5. Oxidative Stress Assessment

Reactive oxygen species production was detected using a cell-permeable non fluorescent probe 2’, 7’- dichlorofluorescin diacetate (DCFH-DA) (KeyGen BioTECH, Nanjing, China). For visualization and quantification of ROS generation in placental villous tissue, flash-frozen villous tissue sections (7 μm) from 7 placentas in each group were incubated with 10 μM DCFH-DA for 20 min at 37 °C, and staining was performed as previously described [22]. Quantification of the fluorescence intensity from dichlorofluorescein (DCF) was performed using the Image J software (NIH Image). For the intracellular level of ROS test, villous cytotrophoblasts were treated with fatty acid (400 μM) or without fatty acid for 48 h. Cells were then harvested, washed with cold PBS, and subjected to DCFH-DA staining in binding buffer at 37 °C for 30 min. Stained cells were analyzed using flow cytometry or a spectrophotometer by measuring DCF fluorescence- activated cell sorting or the fluorescence intensity of DCF at an excitation wavelength of 488 nm and emission wavelength of 530 nm according to the manufacturer’s protocol as previously reported [23].

For the key oxidative enzyme activity detection, placental tissue (100 mg) was homogenized in ice-cold cell and tissue lysis buffer (Beyotime Biotechnology, Nanjing, China) using T18 digital ULTRA-TURRAX Package disperser (IKA, Shanghai, China). The homogenate was centrifuged at 10,000× *g* for 10 min at 4 °C. Then total antioxidant capacity (TAC), superoxide dismutase (SOD) activity, glutathione peroxidase (GSH-PX) activity and catalase (CAT) activity measurements were performed using the commercially available kits from Jiangcheng Bioengineering Institute (Nanjing, China) as previously described [24]. For reduced glutathione (GSH)/oxidative glutathione (GSSG) ratio measurement, the GSH and GSSG Assay Kit (KeyGen BioTECH, Nanjing, China) was used as previously reported [23].

### 2.6. Mitochondrial Analysis

For mitochondrial biogenesis assessment, mitochondrial DNA (mtDNA) copy number was detected by real-time PCR, using the 2^−ΔΔCt^ method as previously described [25]. Total genomic DNA was isolated from placenta of BFI and BFII sows (n = 10 from each group) or cytotrophoblasts treated with fatty acid (400 μM) or not for 48 h in vitro (n = 3 from each group), using TIANamp Genomic DNA Kit (TIANGEN BIOTECH, Beijing, China). A pair of primers (Table S1) for ND1 and Cyclophilin-A were used to amplify a mitochondrial and nuclear DNA fragment, respectively. Citrate synthase (CS) activity was determined in villous tissue or cytotrophoblasts using a commercial kit from Sigma (CS0720; Shanghai, China) according to the manufacturer’s protocol. Placental tissues (200 mg) or cells were homogenized or solubilized in ice-cold extraction buffer, and the homogenate or cell lysate was centrifuged at 16,000× *g* for 20 min at 4 °C. Then CS activity was measured in the supernatant by a spectrophotometer method as previously reported [14].

Fluorescent probe JC-1 (KeyGen BioTECH, Nanjing, China) was used to estimate mitochondrial membrane potential (ΔΨm) as previously described [17,23]. Cytotrophoblasts were harvested, washed with cold PBS, and incubated at 37 °C for 15 min with 5 μg/mL JC-1. After incubation, cells were analyzed by fluorescence- activated cell sorting using flow cytometry. The stained cells with high mitochondrial membrane potential showed red fluorescence and were present in the upper right (UR) quadrant of the FACS histogram.

For mitochondrial complexes activity and ATP production rate measurement, mitochondria was isolated from placental villous tissue or cell homogenate using Mitochondria Isolation Kit (KeyGen BioTECH, Nanjing, China) as previously described [17]. Briefly, placental samples (200 mg) or cytotrophoblasts were homogenized in ice-cold isolation buffer. The homogenate was centrifuged at 1200× *g* for 5 min at 4 °C. The pellet was discarded, supernatant collected and centrifuged at 7000× *g* for 10 min at 4 °C. The resulting supernatant was discarded, and the pellet re-suspended in the ice-cold suspension buffer and centrifuged at 9500× *g* for 5 min at 4 °C. The mitochondria was collected in the sediments. The activities of mitochondrial complexes were determined in isolated mitochondria using the MitoCheck Complex I and II/III Activity Assay Kits (Cayman Chemical Company, Ann Arbor, MI, USA) as previously reported [23]. The mitochondrial ATP production rate was measure using ENLITEN ATP Assay System (Promega; Madison, WI, USA) with 1420 Multilabel Counter (PerkinElmer; Fremont, CA, USA) according to the manufacturer’s instruction.

Mitochondrial respiration of the cultured cytotrophoblasts was measured using a Seahorse XF24 analyzer (Seahorse Biosciences, Shanghai, China) as previously described [26]. Briefly, cells, 2 × 10^6^ total, were suspended in 2 mL DMEM/F12 culture medium and transferred into the chamber, which was maintained at 37 °C. After equilibration and stirring, basal respiration was measured as the average oxygen consumption from four baseline oxygen consumption rates (OCR) readings, and mitochondrial respiratory parameters were calculated from OCR readings following sequential injection of oligomycin (1 μM) and p-trifluoromethoxy carbonyl cyanide phenylhydrazone (FCCP, 2 μM), respectively. OCR was normalized to total cellular protein. Protein concentration was determined using Pierce BCA Protein Assay Kit (Thermo Scientific, Waltham, MA, USA).

### 2.7. Transmission Electron Microscopy

To characterize alterations in the mitochondrial structure and density, placental tissue (small pieces) or cytotrophoblast cultures was fixed in 30 mg/L glutaraldehyde overnight at 4 °C, postfixed in 1% Osmium tetroxide for 1 h at 4 °C, dehydrated, and embedded in Epon-812. The tissue or cell pellet was then cut using an RMC/MTX ultramicrotome (Boeckeler, Tucson, AZ, USA), and ultrathin sections (50 nm) were mounted on copper grids, contrasted with 8% uranyl acetate and lead citrate, and observed with a JEM-1400 Plus transmission electron microscopy (Jeol LTD, Tokyo, Japan). Image analysis was performed using the Image-ProPlus6.0 software from Media Cybernetics (Rockville, MD, USA).

### 2.8. Real-Time Quantitative PCR Analysis

Total RNA was extracted from placental tissue (n = 10 from each group) or cultured cells (n = 3 from each group) with the High Pure RNA tissue kit (Omega Bio-Tek, Norcross, GA, USA) and 500 ng of total RNA was reverse transcribed using PrimeScript RT Master Mix Kit (TaKaRa, Tokyo, Japan). Primers (Table S1) were synthesized by Invitrogen (Shanghai, China). Quantitative PCR was conducted on the Step One Plus Real-Time PCR System (ABI, Waltham, MA, USA) with the following program: 95 °C for 30 s, 95 °C for 5 s, 60 °C for 30 s, 95 °C for 15 s, 60 °C for 1 min, and 95 °C for 15 s, with 40 cycles of steps 2 and 3. Amplification was performed in 25 μL reaction system containing specific primers (Table S1) and SYBR Premix Ex Taq II (TaKaRa, Tokyo, Japan). Relative gene expression was calculated using the comparative Ct method with the formula 2^-ΔΔCt^ [27]. The two reference genes GAPDH and HPRT1 were used. The geometric mean of relative gene expression was calculated and used for further analysis as previously reported [28].

### 2.9. Protein Carbonylation and Western Blotting Analysis

Protein carbonyl derivatives (a marker of oxidative stress) were detected using OxiSelect Protein Carbonyl Immunoblot Kit (Cell Biolabs, San Diego, CA, USA) as previously reported [5]. Briefly, after the derivatization of the protein sample on PVDF with dinitrophenylhydrazine (DNPH) following the manufacturer’s instructions, the blot was developed using a standard immunoblot protocol. Total protein from cultured trophoblast cells was extracted using cell lysis buffer (Beyotime Biotechnology, Nanjing, China) by procedures as previously described [29]. Mitochondrial fractions for Western blotting were obtained from placental villous tissue using Mitochondrial Protein Extraction Kit (KeyGen BioTECH, Nanjing, China) and suspended in lysis buffer supplemented with a protease and phosphatase inhibitor cocktail (Sigma, Saint Louis, MO, USA). Protein samples (50 μg) were separated by SDS-PAGE and transferred to PVDF membrane (Bio-Rad Laboratories, Hercules, CA, USA). After blocking in 5% fat-free milk for 1 h at room temperature, the membranes were incubated with rabbit anti-Mark4 (4834, dilution 1:1000, Cell Signaling Technology, Danvers, MA, USA), AMPK (5831, dilution 1:1000, Cell Signaling Technology), Phospho-AMPK (2535, dilution 1:1000, Cell Signaling Technology), Phospho-NF-κB (3033, dilution 1:1000, Cell Signaling Technology), GAPDH (2118, dilution 1:1000, Cell Signaling Technology), NF-κB (ab90532, dilution 1:1000, Abcam Biotechnology, Cambridge, MA, USA), ATP5α (ab245580, dilution 1:2000, Abcam Biotechnology) and Phospho-Mark4 (SAB4504258, dilution 1:500, Sigma, Saint Louis, MO, USA) antibody, or Mouse anti- NDUFB8 (ab110242, dilution 1:1000, Abcam Biotechnology), SDHB (ab14714, dilution 1:2000, Abcam Biotechnology), and UQCRC2 (ab14745, dilution 1:2000, Abcam Biotechnology) antibody overnight at 4 °C, followed by incubation with Goat anti- mouse or rabbit IgG horseradish peroxidase (HRP)-conjugated secondary antibodies (HAF007 and HAF008, dilution 1:2000, RD SYSTEMS, Minneapolis, USA) for 1 h at room temperature. Proteins were visualized using the LumiGLO Reagent and Peroxide system (Cell Signaling Technology, Danvers, USA), and then the blots were quantified using Bio-Rad ChemiDoc imaging system (Bio-Rad Laboratories, Hercules, USA). Band density was normalized according to the GAPDH content.

### 2.10. Statistical Analysis

Statistical analyses were conducted using SPSS Statistics 20.0 software (IBM SPSS, Armonk, NY, USA). Data obtained from sows and placentas were analyzed as a completely randomized design. Each sow and her litter were considered as an experimental unit. Statistical differences between groups were evaluated using Independent-Samples T Test. For all cell culture experiments, data were obtained from at least three independent experiments. Statistical differences among individual means were analyzed using One-way ANOVA for comparisons among groups, followed by Duncan test. Results were expressed as means ± SEM. A *p*-value < 0.05 was considered statistically significant, and very significant was indicated when *p* < 0.01.

## 3. Results

### 3.1. Characteristics of the Studied Sows

BFII sows had markedly greater body weight (BW) and BF than BFI sows (*p* < 0.05), at mating (BW: 173.18 ± 0.52 vs. 167.70 ± 0.30 kg; BF: 24.11 ± 0.33 vs. 16.51 ± 0.21 mm) and at farrowing (BW: 221.22 ± 0.43 vs. 214.38 ± 0.28 kg; BF: 25.09 ± 0.31 vs. 18.63 ± 0.18 mm), respectively (Table 1). The litter size, litter live size, litter weight and placental efficiency were lower in BFII sows than those in BFI group (*p* < 0.05), whereas both groups were similar in terms of birth weight and placental weight. Consistent with increased adiposity, there were 47% and 76% increases (*p* < 0.05) in TG and NEFA levels in maternal plasma from BFII sows, respectively, compared to the BFI group (Table 1). Excessive back-fat was also associated with higher leptin levels (*p* < 0.05) in maternal plasma. Concurrently, we observed increased H_2_O_2_ levels in BFII sows compared with BFI group (*p* < 0.05).

### 3.2. Excessive Back-Fat is Associated with Increased Placental Oxidative Stress

**Figure 1:**
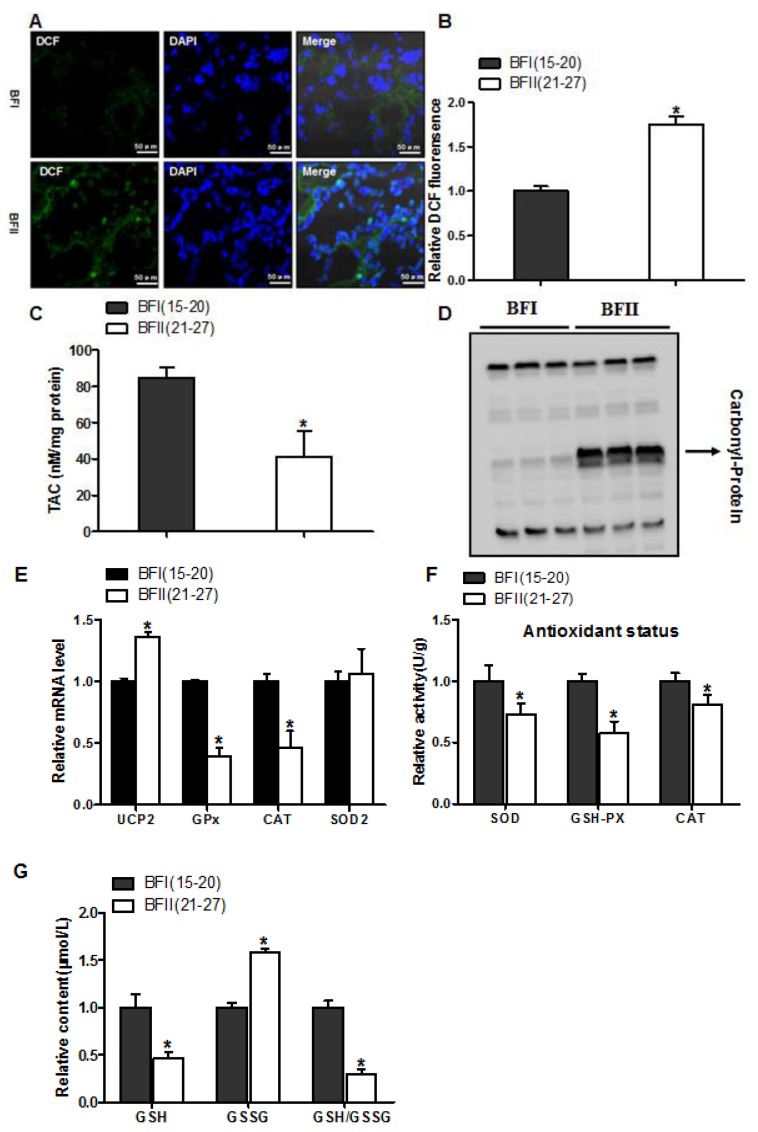
Increased oxidative stress in placentas with increasing back-fat. Representative visualization (**a**) and quantification (n = 7 in each group) (**b**) of dichlorofluorescein (DCF) in cryosections of placenta. Scale bar: 50 μm; (**c**) Total antioxidant capacity (TAC) was measured in term placentas from BFI and BFII sows (n = 10/group). TAC was normalized to total protein level and expressed as nM TAC per mg protein; (**d**) Representative immunoblots showing total protein carbonylation in placentas of BFI and BFII sows; (**e**) mRNA levels of oxidant stress-related genes determined by real-time RT-PCR in placentas from BFI and BFII sows (n = 10/group); (**f**) Placental antioxidant status was estimated by SOD, GSH-PX, and CAT (n = 10/group); (**g**) Relative content of GSH/GSSG in placentas from BFI and BFII sows (n = 10/group). Results were expressed as fold change versus the BFI sow set to 1 unit. Values are expressed as mean ± SEM. * *p* < 0.05 compared with the BFI group. DCF: dichlorofluorescein; SOD: superoxide dismutase; GSH-PX: glutathione peroxidase; CAT: catalase; GSH: reduced glutathione; GSSG: oxidative glutathione; BF: back-fat thickness.

Considering BFII sows had higher plasma H_2_O_2_ levels (an indicator of systemic oxidative stress), we investigated the impact of excessive back-fat on oxidative stress in the placenta of sows. Oxidant status alterations in BFII placenta were marked by higher production of ROS assessed by DCF staining, decreased placental TAC and elevated protein carbonylation (a marker of protein oxidation), compared to the BFI group (*p* < 0.05, Figure 1A–D). The mRNA level of uncoupling protein 2 (UCP2, a marker of increased mitochondrial ROS production) was induced in the placenta of BFII sows, but the mRNA expression of genes associated with antioxidant system, including glutathione peroxidase (GPx) and catalase (CAT), was decreased in BFII group compared with BFI group (*p* < 0.05, Figure 1E), without a significant change in superoxide dismutase 2 (SOD2). Consistent with increased oxidative stress, the antioxidant enzyme activities of superoxide dismutase, glutathione peroxidase and catalase were decreased in BFII group (*p* < 0.05, Figure 1F). In addition, in the placenta of BFII sows, a decrease (*p* < 0.05) was noted in glutathione (GSH)/oxidative glutathione (GSSG) ratio compared to values obtained with placentas from BFI sows (Figure 1G). Thus, we concluded excessive back-fat was associated with increased oxidative stress in the full-term pig placenta.

### 3.3. Effect of Excessive Back-Fat on Placenta Mitochondrial Oxidative Respiration

**Figure 2:**
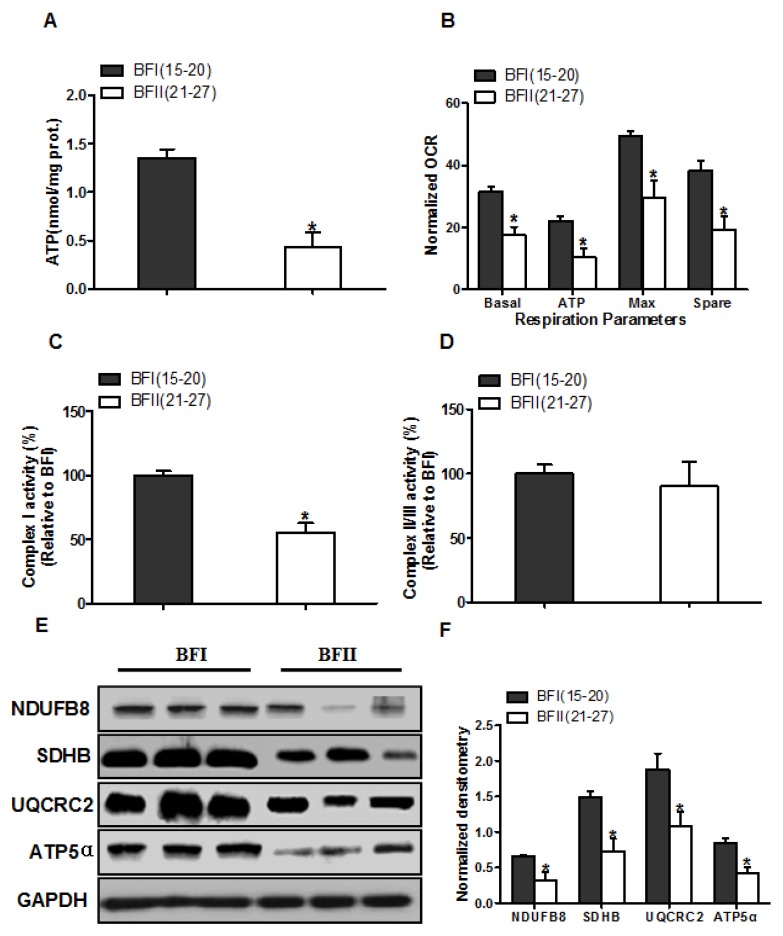
Impairment of placental mitochondrial oxidative respiration in sow with increased back-fat. (**a**) Mitochondrial ATP production in placental tissue from BFI and BFII sows. ATP levels were normalized to total protein level of villous tissue extract; (**b**) Mitochondrial respiratory parameters were measured in cytotrophoblast cultures isolated from placentas of BFI and BFII sows. Before assessment of mitochondrial respiration, cytotrophoblasts were incubated with 400 μM NEFA for 24 h. Oxygen consumption rate (OCR) measurements were normalized to total protein content (pmol O_2_/μg protein); (**c** and **d**) Placental mitochondrial complexes I (**c**) and combined II/III (**d**) activity in BFI and BFII sows; (**e**) Representative immunoblot analysis of mitochondrial complexes I (NDUFB8), II (SDHB), III (UQCRC2) and V (ATP5α) in placental mitochondrial fractions from BFI and BFII sows; (**f**) Densitometric analysis of corresponding proteins in E by normalization to GAPDH as an internal control. Results are expressed as mean ± SEM. * *p* < 0.05 compared with the BFI group. n = 10 in each group. BF: back-fat thickness; NEFA: non-esterified fatty acid; Basal: basal respiration; ATP: ATP-coupled respiration; Max: maximal respiration; Spare: spare capacity.

Because ROS is mainly generated from mitochondria, we subsequently tested whether an increase in production of ROS is associated with mitochondrial dysfunction in placenta of BFII sows. We initially evaluated mitochondrial function by detecting the levels of ATP in placental tissue. As illustrated in Figure 2A, the ATP content was lower (68% decrease, *p* < 0.05) in the BFII placentas compared with BFI group. To determine whether changes in placental ATP levels are related to altered mitochondrial respiration, we next investigated the effects of excessive back-fat on mitochondrial function in vitro using cultured cytotrophoblasts (Figure 2B). Cytotrophoblasts from BFII sows cultured in fatty acid demonstrated a significant decrease in basal mitochondrial oxygen consumption, ATP-coupled respiration, maximal respiration and spare capacity compared with cells from BFI group (*p* < 0.05, Figure 2B), suggesting that mitochondrial function was compromised in the placenta of sows with excessive back-fat. Consistent with reduced mitochondrial oxidative respiration, the activity of mitochondrial Complex I was also decreased in BFII group (*p* < 0.05; Figure 2C), whereas there was no difference in coupled mitochondrial Complex II/III activity (Figure 2D). To explore the mechanism that may account for the reduction in placental mitochondrial respiration with increasing back-fat, we utilized a immunoblot approach to measure changes in protein expression of the mitochondrial electron transport complexes. As shown in Figure 2E,F, the expression levels of subunits of complexes I (NDUFB8), II (SDHB), III (UQCRC2), and V (ATP5α) were significantly reduced (*p* < 0.05) in placentas of BFII sows compared with BFI sows.

### 3.4. Effect of Excessive Back-Fat on Placental Mitochondrial Content

**Figure 3:**
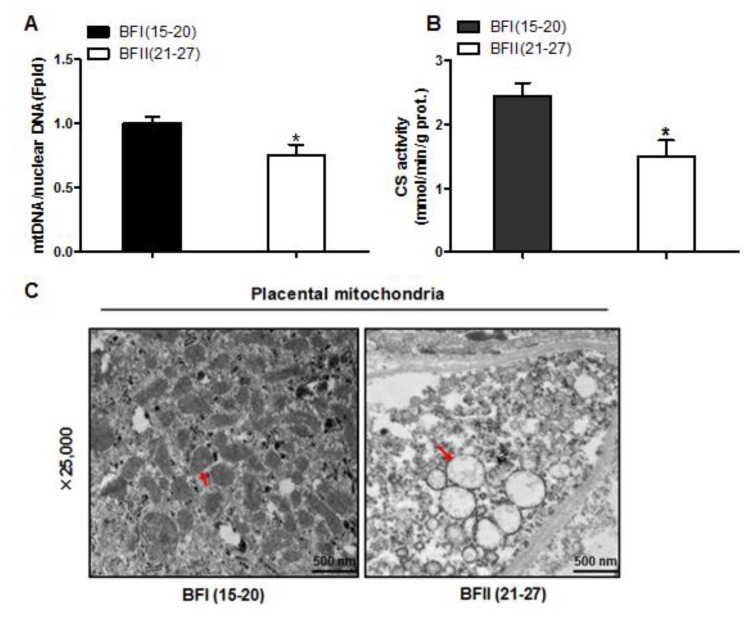

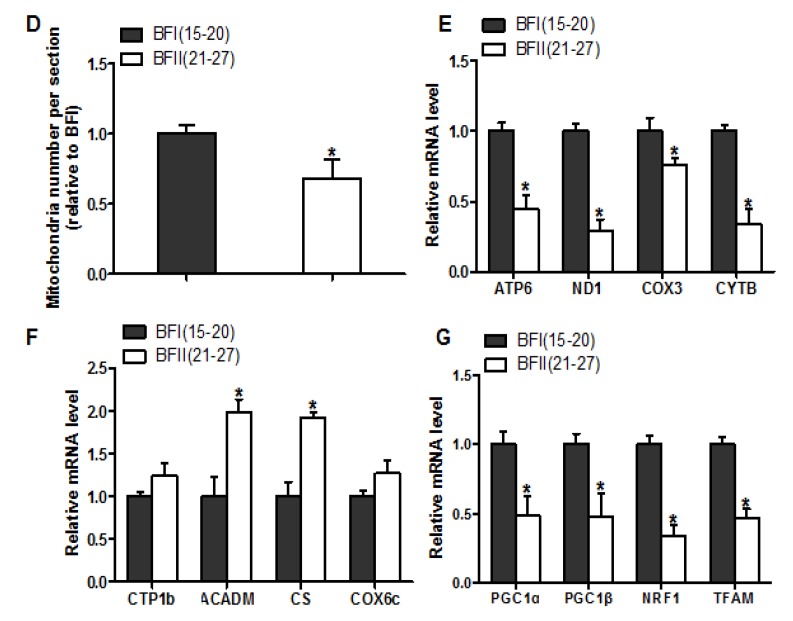
Decreased mitochondrial density in the placenta of sow with excessive back-fat. Mitochondrial biogenesis was estimated by bothmtDNA copy number (**a**) and CS activity (**b**) in the placenta from BFI and BFII sows (n = 10/group); (**c**) Mitochondrial density assessed by electron microscopy in the placentas of BFI and BFII sows. Original magnification, 25,000×; (**d**) Quantification of mitochondrial number per image area in whole placental villous tissue from 7-BFI and 7-BFII sows (analysis of 10 random images per placenta). (**e**–**g**) The relative mRNA level of various mitochondria-encoded genes (**e**), nuclear-encoded mitochondrial genes (**f**), and mitochondrial biogenesis genes (**g**) was determined by quantitative RT-PCR in placentas from BFI and BFII sows (n = 10/group). Results were normalized by the mean value for the BFI sow set to 1 unit. Values are expressed as mean ± SEM. * *p* < 0.05 compared with the BFI group. BF: back-fat thickness; mtDNA: mitochondrial DNA; CS: citrate synthase; Red arrow: mitochondria.

We next investigated the effects of excessive back-fat on placenta mitochondrial density. As shown in Figure 3A, the ratio of mtDNA to nuclear DNA in the placenta was significantly more decreased in the BFII sows than in the BFI sows (*p* < 0.05). In agreement, mitochondrial content estimated by citrate synthase (CS) activity showed a significant reduction in placentas of BFII sows (*p* < 0.05) compared with BFI group (Figure 3B). Using transmission electron microscopy, we found that the amount of mitochondria in placental villi was lower in the BFII sows (32% decrease, *p* < 0.05) than in the BFI group (Figure 3C,D). Consistent with reduced mitochondrial DNA (mtDNA) copy number and CS activity, the mRNA levels of mitochondria-encoded genes, including ATP6, ND1, COX3, and CYTB, were lower in placenta of BFII sows (*p* < 0.05, Figure 3E), whereas the mRNA content of several nuclear-encoded mitochondrial genes, including ACADM and CS, was increased in the placenta of BFII sows (*p* < 0.05) compared with BFI group (Figure 3F), without a significant change in CTP1b and COX6c. To clarify the mechanisms implicated in the reduction of mitochondrial density in the placenta of BFII sows, we measured the mRNA levels of genes involved mitochondrial biogenesis. As illustrated in Figure 3G, the mRNA expression of PGC1α, PGC1β, nuclear respiratory factor 1 (NRF1), and mitochondrial transcription factor A (TFAM) was lower in the placenta of BFII sows than that of BFI sows (*p* < 0.05).

### 3.5. Alteration of Mitochondrial Ultrastructure in the Placenta of Sows with Excessive Back-Fat

**Figure 4:**
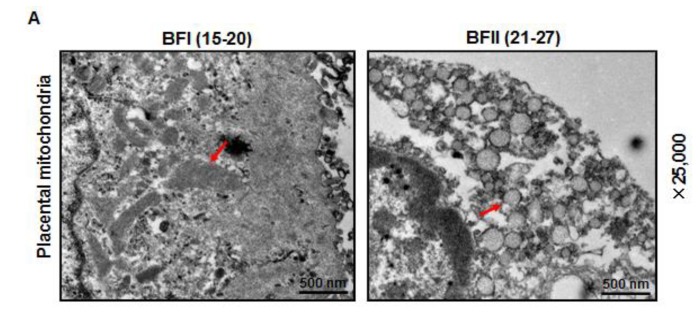

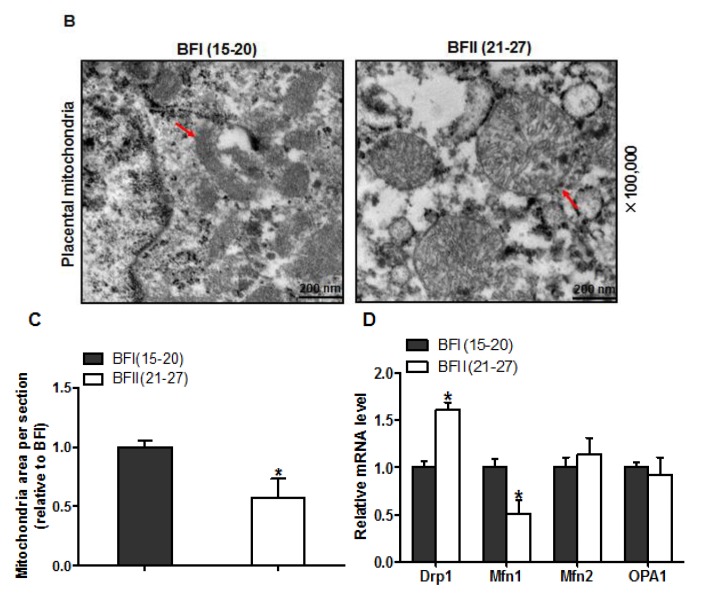
Alterations in the mitochondrial structure of placenta of sow challenged with excessive back-fat. (**a** and **b**) Transmission electron microscopy images at original magnifications of 25,000× (**a**) and 100,000× (**b**) in placental mitochondria from BFI and BFII sows; (**c**) Quantification of mitochondria area in the placental villi sections from 7-BFI and 7-BFII sows (analysis of 10 random images per placenta). Results were normalized by the mean value for the BFI sow set to 1 unit; (**d**) mRNA levels of mitochondrial fission- and fusion-related regulators, determined by quantitative RT-PCR, in placentas from BFI and BFII sows (n = 10/group). Values are expressed as mean ± SEM. * *p* < 0.05 compared with the BFI group. BF: back-fat thickness; Red arrow: mitochondria.

In addition to the observed reduction in mitochondrial content, the transmission electron microscopy analysis revealed marked alterations in mitochondrial morphology in the placenta of BFII sows. As shown in Figure 4A,C, the area of mitochondria was decreased (43% reduction, *p* < 0.05) in placenta from BFII sows compared to BFI group. Higher magnification (100,000×) demonstrated swelling of mitochondria associated with an increased number of disarrayed cristae and a decreased electron density of the matrix in the placenta of BFII sows (Figure 4B). Concerning mitochondrial morphology regulation, we investigated the mRNA content of genes associated with mitochondrial fission and fusion, including Dynamin 1 (Drp1), mitofusin 1 (Mfn1), mitofusin 2 (Mfn2), and optic atrophy type 1 (OPA1), which play a key role in this process [30]. As illustrated in Figure 4D, excessive back-fat induced an increase (*p* < 0.05) in Drp1 (regulating mitochondrial fission) mRNA level in placenta, whereas reduced mRNA expression of Mfn1 (regulating mitochondrial fusion) was observed in the placenta from BFII sows (*p* < 0.05). Both Mfn2 and OPA1 (regulating mitochondrial fusion) expression were not affected by excessive back-fat.

### 3.6. ROS Induce Mitochondrial Alterations and Dysfunction in Cultured Cytotrophoblasts

**Figure 5:**
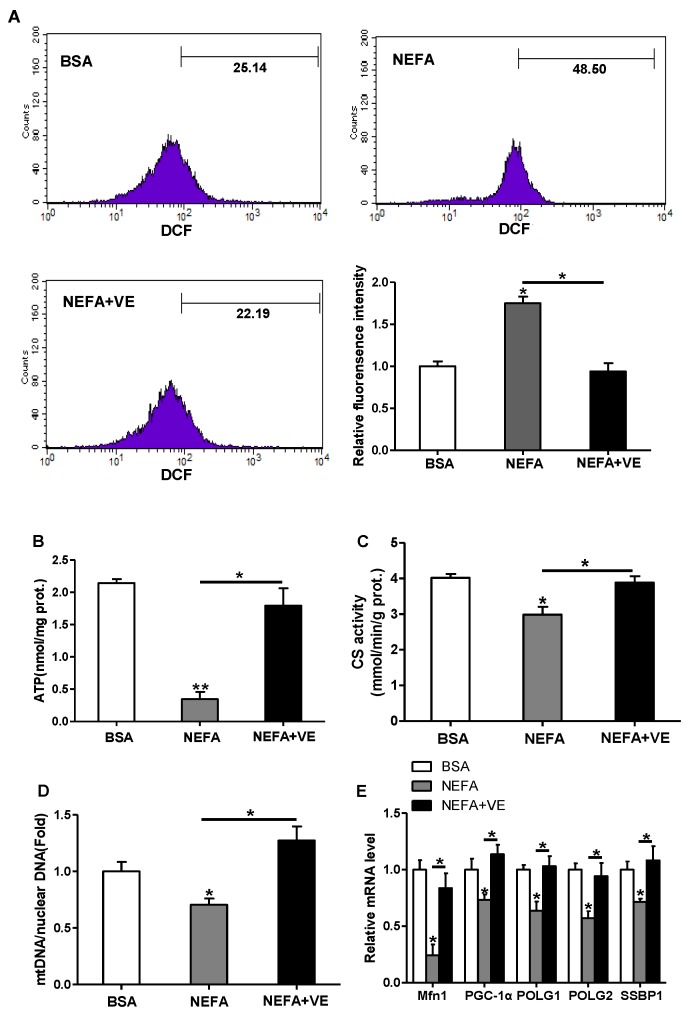
ROS-induced mitochondrial alterations in pig primary trophoblast cells challenged with 400 μM NEFA. (**a**) ROS generation, measured by DCF production using flow cytometry or a spectrophotometer (n = 3), respectively, in cytotrophoblasts isolated from placenta of BFI sows. Before assessment of ROS production, cells were incubated with 400 μM NEFA in the presence or absence of 2 mM VE for 48 h. Value in scale bar depicts the percentage of DCF-positive cells; (**b**) Mitochondrial ATP production in cytotrophoblasts incubated with 400 μM NEFA in the presence or absence of 2 mM VE for 48 h (n = 3). ATP levels were normalized to total protein level of whole cell lysates extract; (**c** and **d**) Mitochondrial biogenesis was assessed by both citrate synthase activity (**c**) and mtDNA copy number (**d**) in cytotrophoblasts incubated with 400 μM NEFA in the presence or absence of 2 mM VE for 48 h (n = 3); (**e**) Relative mRNA expression of genes implicated in mitochondrial biogenesis and mtDNA replication (n = 3). Results were expressed as fold change relative to the values of untreated cells (BSA treatment) set to 1 unit. Values are expressed as mean ± SEM. * *p* < 0.05; ** *p* < 0.01 compared with the control group. ROS: reactive oxygen species; DCF: dichlorofluorescein; VE: Vitamin E; mtDNA: mitochondrial DNA; BSA: bovine serum albumin; NEFA: non-esterified fatty acid; Control: BSA group.

Since exposure to lipid oversupply leads to increased mitochondrial ROS production in a variety of peripheral tissues, we examined the effects of high lipid level on ROS production and mitochondrial density and functions in pig placental trophoblasts. ROS production was markedly induced by fatty acid (400 μM) treatment for 48 h (DCF: 48.5% in NEFA vs. 25.14% in BSA), and the addition of VE (2 mM) blocked this effect (*p* < 0.05, Figure 5A). Incubation with fatty acid also decreased ATP content (Figure 5B), CS activity (Figure 5C), and mtDNA level (Figure 5D). Furthermore, the mRNA levels of Mfn1, PGC1α, and genes implicated in mtDNA replication and repair, including gamma DNA polymerase subunit 1 (POLG1) and 2 (POLG2) and single-strand DNA binding protein 1 (SSBP1), were decreased in cytotrophoblasts treated with fatty acid for 48 h (Figure 5E). Concurrently, JC-1 fluorescent staining analysis showed mitochondrial membrane potential, which represented the oxidative respiration level, was reduced in fatty acid treatment (UR: 56.59% in NEFA vs. 89.89% in control, Figure S1A). The addition of VE counteracted all these effects, which indicated that ROS contributed to the observed mitochondrial alterations and dysfunction in cultured cytotrophoblasts (Figure 5B–E and Figure S1A). Transmission electron microscopy study nicely illustrated that the addition of fatty acid for 48 h negatively altered mitochondrial structure in cytotrophoblasts compared with control cells (Figure S1B).

In order to further reveal the mechanisms of ROS induced mitochondrial alterations in cytotrophoblasts, western blot analysis of key regulators of mitochondrial function was performed. As shown in Figure S1, microtubule affinity regulating kinase 4 (Mark4), phos-Mark4 (Thr 214), and phos- NF-κB (Ser536) were up-regulated (*p* < 0.05) in cytotrophoblasts incubated with fatty acid for 48 h, compared to control cells (Figure S1C). In contrast, the expression of phos-AMPK (Thr 172) was decreased in fatty acid treatment compared with control group (*p* < 0.05, Figure S1D). Moreover, the addition of VE alleviated these effects (Figure S1D). The protein content of AMPK and NF-κB was not affected by the addition of fatty acid (Figure S1C,D).

## 4. Discussion

**Figure 6:**
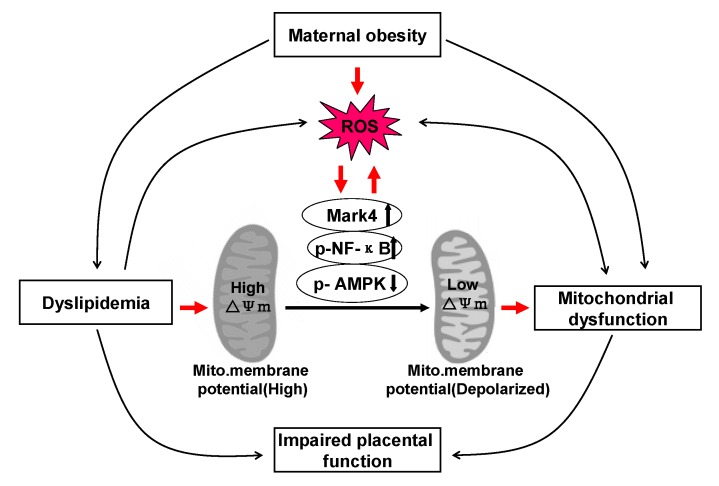
A schematic diagram depicts a causative role of obesity-induced oxidative stress in mitochondrial injury and placental dysfunction. Excessive back-fat aggravates dyslipidemia (lipotoxicity), which induces oxidative stress and mitochondrial dysfunction in the full-term pig placenta. Increased back-fat promotes mitochondrial oxidative injury by activating Mark4 and NF-κB and reducing AMPK activation. ↑: up-regulation of gene expression. ↓: down-regulation of gene expression. Arrows indicates a positive regulation. Interactions depicted are based on studies performed in various tissues (in some cases placenta) and have been previously published. ROS: reactive oxygen species.

As a mitochondrial rich organ, the placenta is essential for the normal growth and development of fetus. Thus, abnormalities in mitochondria may have detrimental consequences on placental function and therefore fetal growth and development. Indeed, cumulative evidence strongly suggests that alterations in mitochondrial respiration function and enzymatic activity of mitochondrial complexes in the human placenta are associated with pregnancy loss complicated by maternal obesity [14,31]. However, whether these changes are correlated with excessive back-fat of sows during pregnancy is not clear. Here, we investigated the influences of excessive back-fat on the amount, structure, and function of mitochondria in the porcine placenta. Our data indicate that mitochondrial defects were evident in term placenta from BFII sows. Furthermore, we found that increased oxidative stress in pig placenta due to excessive back-fat is probably one of the major determinants of mitochondrial alterations. This is supported by data showing that (a) an increase in ROS production occurred specifically in the placenta of BFII sows; (b) excessive ROS production was also associated with mitochondrial abnormalities in placenta from BFII sows; (c) incubation of cultured trophoblast cells with high lipid concentration induced ROS production and altered mitochondrial density and functions; and (d) these effects were alleviated by antioxidant treatment. Together, these findings suggest excessive back-fat aggravates a lipotoxic placental milieu (summarized in Figure 6) that induces oxidative stress and mitochondrial dysfunction in the full-term pig placenta.

In this study, excessive back-fat of sows was associated with increased plasma lipid and leptin levels, which indicated that the BFII sows had the trend for a higher adiposity than BFI sows. These metabolic alterations were associated with increased systemic (elevated plasma H_2_O_2_ level) and placenta (high levels of placental protein carbonylation and GSSG) oxidative stress from BFII sows compared to BFI sows, probably because of an increase in placental ROS production and a reduction in antioxidant defenses (decreased activities of SOD, GSH-PX, and CAT) in placenta of BFII sows. Furthermore, these disturbances were associated with mitochondrial changes in term placenta of sows with excessive back-fat. There was a significant decrease in placental ATP content associated with reduced expression levels of subunits encoding the complexes of mitochondrial electron transport chain (complexes I, II, III, and V), indicating a mitochondrial dysfunction in the placenta from BFII sows compared with BFI group. This observation agrees with previous reports in obese placenta of human model [14,15]. Since placenta is an extremely metabolically active tissue with rich energy-producing mitochondria, it is conceivable that mitochondrial abnormalities in ATP production may be not able to support placental substantially large energy requirements, therefore negatively affecting placental function and fetal development [14]. Consistent with this notion, a reduction in litter weight and placental efficiency was observed in BFII sows, which suggested that the placentas from BFII sows failed to support proper fetal development. Although a significant difference in birth weight between BFII and BFI groups was not observed, it is noted that a sexually dimorphic adaptation of the placenta may contribute to the differences in fetal growth and survival responding to the adverse intrauterine environment [32]. Recently, it has been demonstrated that pro-inflammatory cytokine production in placenta and the effect of inflammation on placental mitochondrial function exist a sexually dimorphic response [33]. Hence, the possibility that a sexual dimorphism may contribute to the lack of difference in the data cannot be completely ruled out. Further studies to assess the consequence of sexual dimorphic responses in placental mitochondrial energetics and function during porcine pregnancy associated with excessive back-fat are warranted.

To further explore the effect of excessive back-fat on placental mitochondrial respiration, we utilized an in vitro model of pig trophoblast cell culture. It is important that isolated trophoblasts can retain their in vivo phenotype in culture without the influence of the other different cells types (e.g., immunocytes and vascular cells) in whole placenta on the results of mitochondrial energy metabolism. Consistent with previous studies in human placenta and other tissues [5,15], we found a reduction in mitochondrial oxidative respiration with increased back-fat. The decreased mitochondrial maximum respiration and spare respiratory capacity reveals that cytotrophoblasts from placenta of BFII sows have a compromised cellular ability to meet energetic needs, and such a decrease in energetic metabolism may render them more susceptible to cellular stressors like lipids and ROS [34]. Measurement of activities of the mitochondrial respiratory complexes is commonly used as markers of mitochondrial function [35]. Consistent with reduced mitochondrial respiration, we observed that there was a significant decrease in mitochondrial complex I activity in term placenta of BFII sows compared to BFI group. As complex I of the mitochondrial respiratory chain is particularly prone to damage by oxidative stress [36], its reduction in activity associated with increased back-fat may be explained by a significant increase in ROS production that was also seen in the placenta of BFII sows. Indeed, increased local concentrations of ROS near the electron transport chain components are believed to be potentially high, making the mitochondrial respiratory complexes themselves likely targets of ROS impairment [13]. However, the activity of coupled complexes II and III was not significantly different between BFII and BFI sows. In contrast to our findings, Hastie et al. (2014) found a significant increase in the activity of placental combined complexes II and III associated with maternal obesity [14]. Differences in placental mitochondria collection may explain the discrepancy in our results. Of note, the mitochondrial inner membrane contains significantly higher complex I than any of the other complexes [36]. Thus, damage to mitochondrial complex II and III would have lesser impact on mitochondrial respiration than damage to complex I.

There is currently growing amounts of evidence showing that, as well as mitochondrial function, mitochondrial content is also altered in the human term placenta associated with maternal obesity [14,15]. Consistent with our expectation, we found that there was a significant decrease in mitochondrial number associated with a reduction in mtDNA content and CS activity (quantitative indexes of mitochondrial content) in placenta from BFII sows compared with BFI sows. These findings were further confirmed by reduced expression levels of mitochondria-encoded genes (ATP6, ND1, COX3, and CYTB) rather than increased mRNA contents of nuclear-encoded mitochondrial genes (ACADM and CS), which suggested that the control of mitochondrial biogenesis is altered in the placenta of BFII sows. Mitochondrial biogenesis is controlled by multiple transcription factors including PGC-1α, PGC-1β, NRF1, TFAM, TFB1M, and TFB2M [30]. Several studies have linked mitochondrial biogenesis transcription factors to placental mitochondrial content or pathology [37,38]. In agreement, we found that PGC-1α, PGC-1β, and downstream targets of PGC-1α, such as NRF1 and TFAM were down-regulated in term placenta of BFII sows compared to BFI group. Furthermore, a striking phenotype of placenta in BFII sows resided in the structural abnormalities of the mitochondria, as revealed by electron microscopy. A number of mitochondria appeared swollen, with fewer cristae, and the inner or outer membranes were sometimes disrupted in the placenta of sows with excessive back-fat. It should be noted that the regulation of mitochondrial dynamic network (maintaining normal mitochondrial structure) occurs through mitochondrial biogenesis and continuous cycles of fission and fusion [39]. In this trial, we observed, however, there was a significant decrease in the mitochondrial fusion regulator (Mfn1) associated with increased expression of Drp1, a protein participating in the mitochondrial fission, which suggested that aggravated mitochondrial fission as a mechanism may contribute to decreased placental mitochondrial content in BFII sows, potentially by disruption of mitochondrial structures [40]. Although our data pointed out that excessive back-fat was associated with mitochondrial alterations in the porcine placenta, further experiments are needed to determine the mediators of placental mitochondrial dysfunction.

To confirm the implication of oxidative stress in placental mitochondrial alterations associated with excessive back-fat, our in vitro data in cultured placental trophoblast cells demonstrated that treatment with high fatty acid concentration induced ROS production and mitochondrial damage in cytotrophoblasts, in agreement with previous reports that elevated lipids can induce cellular dysfunction through the activation of inflammation and the overproduction of ROS causing mitochondrial dysfunction in various models [6,41]. Although we were not able to determine whether ROS production is the only factor contributing to mitochondrial dysfunction, it is noticed that oxidative stress induced by lipid resulted in a decrease in the amount of mtDNA and CS activity in cultured cytotrophoblasts, mitochondrial abnormalities in function (reduced ATP production and mitochondrial membrane potential) and structure (mitochondrial swelling and disruption), and a concomitant reduction in expression of genes associated with mitochondrial biogenesis (PGC-1α and Mfn1) and mtDNA replication (POLG1, POLG2, and SSBP1), and these effects were reversed by antioxidant treatment, supporting a critical role of ROS in mediating mitochondrial alterations in placenta of sows with excessive back-fat. In addition, obese pregnancy has been previously linked to a heightened inflammatory state in placenta of human beings and animals such as pigs, with significant increases in pro-inflammatory cytokines such as TNFα, IL-6, and IL-8, which may impair mitochondrial function through activation of NF-κB signaling pathway [33]. To further estimate the mechanism of ROS-induced mitochondrial dysfunction in the term placenta of BFII sows, we initially investigated the status of activation of inflammatory NF-κB signaling in in vitro model of pig trophoblast cells challenged with high lipid. Consistently, we found that elevated lipid promoted the activation of NF-κB associated with increased ROS and impaired mitochondrial function and structure in cytotrophoblasts. Moreover, our previous studies showed that excessive back-fat is associated with increased activation of Mark4 and reduced activity and expression of AMPK in pig term placenta, indicating a potential mechanism for increased placental inflammation and oxidative stress [8,17]. In this trial, increased activation of microtubule affinity-regulating kinase 4 (Mark4) and reduced activation of AMPK were also observed in cultured trophoblast cells treated with high fatty acid, and addition of vitamin E alleviated these effects, suggesting that Mark4 and AMPK may be involved in regulation of ROS-mediated mitochondrial abnormalities in pig placenta induced by increased back-fat. In agreement with this conclusion, it has been shown that Mark4 promotes mitochondrial oxidative injury and adipose inflammation via activating NF-κB signal pathway [23]. Furthermore, studies have identified that AMPK activation could prevent inflammatory signaling pathways [1] and increases the expression of genes involved in mitochondrial function in human or mice skeletal muscle [42,43]. However, the precise molecular mechanisms by which these regulators improve or impair mitochondrial function in porcine placenta associated with excessive back-fat need to be further studied.

## 5. Conclusions

In summary, the present study demonstrates that excessive back-fat incites placental mitochondrial alterations that correspond with reduced ATP content and mitochondrial dysfunction in the pig full-term placenta. Moreover, augmented mitochondrial abnormalities may be attributed to increased placental oxidative stress in conditions associated with elevated maternal circulating lipids such as excessive back-fat during pregnancy of sows, and increased NF-κB signaling may contribute to ROS-mediated mitochondrial dysfunction in pig placenta induced by increased back-fat. Thus, our results suggest that excessive back-fat-induced oxidative stress and subsequent mitochondrial dysfunction has potential as a causal mechanism to impact pig placental function and therefore impair fetal growth and development.

## Figures and Tables

**Table 1:** Characteristics of pregnancies for sows studied ^†^.

	BFI (15–20 mm, n = 10)	BFII (21–27 mm, n = 10)
Parity	2	2
BF at mating, mm	16.51 ± 0.21 ^a^	24.11 ± 0.33 ^b^
BW at mating, kg	167.70 ± 0.30 ^a^	173.18 ± 0.52 ^b^
BF at farrowing, mm	18.63 ± 0.18 ^a^	25.09 ± 0.31 ^b^
BW at farrowing, kg	214.38 ± 0.28 ^a^	221.22 ± 0.43 ^b^
TG *, mg/dL	21.15 ± 2.14 ^a^	31.18 ± 1.36 ^b^
NEFA *, mmol/L	0.21 ± 0.04 ^a^	0.37 ± 0.03 ^b^
Leptin *, ng/mL	13.13 ± 1.20 ^a^	20.02 ± 2.31 ^b^
H_2_O_2_ *, μM/L	66.20 ± 3.71 ^a^	89.32 ± 3.01 ^b^
Litter size ^#^	14.83 ± 0.31 ^a^	12.88 ± 0.53 ^b^
Litter live size	12.75 ± 0.35 ^a^	11.23 ± 0.86 ^b^
Placental weight, kg	3.22 ± 0.19	3.38 ± 0.25
Birth weight ^‡^, kg	1.42 ± 0.05	1.38 ± 0.23
Litter weight, kg	18.04 ± 0.23 ^a^	15.42 ± 0.75 ^b^
Placental efficiency ^§^	5.59 ± 0.16 ^a^	4.61 ± 0.22 ^b^

BF, Back-fat thickness; BW, Body weight; TG, triglyceride; NEFA, non-esterified fatty acid; ^†^: Results are expressed as means ± SEM. Means within the same row with different superscripts mean significant difference (*p* < 0.05); *: A value measured in maternal plasma; #: The number of live-born and stillborn piglets in litter; ^‡^: Birth weight of born alive and stillborn piglets; ^§^: A ratio of litter weight to placental weight.

## Supplementary Materials

The following are available online at https://www.mdpi.com/2076-2615/10/2/360/s1. Figure S1: Effects of ROS on mitochondrial structure and function in pig primary trophoblast cells. Table S1: Primer sets used for real-time PCR.
